# Unveiling the ecological dominance of button mangrove (*Conocarpus erectus* L.) through microstructural and functional traits modifications across heterogenic environmental conditions

**DOI:** 10.1186/s40529-024-00440-0

**Published:** 2024-11-29

**Authors:** Ummar Iqbal, Akkasha Azam, Khawaja Shafique Ahmad, Sahar Mumtaz, Ansar Mehmood, Nargis Naz, Zartasha Usman, Hina Abbas, Muhammad Akram

**Affiliations:** 1 Department of Botany, The Islamia University of Bahawalpur, Bahawalpur, 63100 Pakistan; 2 Department of Botany, University of Poonch Rawalakot, Rawalakot, 12350 Pakistan; 3 Department of Botany, University of Education, Vehari Campus, Punjab, 61100 Pakistan

**Keywords:** Adaptive strategies, Button Mangrove, Osmoregulation, Succulence, Xeromorphy

## Abstract

**Background:**

The button mangrove (*Conocarpus erectus* L.) is regarded as a peripheral species within mangrove communities. This particular species has the ability to thrive in regions that are arid or semiarid, where there is limited availability of nutrients. This study provides evidence of the ecological dominance of *Conocarpus erectus* across various habitats, highlighting its adaptability and success throughout the country of Pakistan. We collected twelve populations from four distinct ecological regions, including artificial forest plantations, agricultural fields, roadsides, and wastelands, offering a comprehensive assessment of *C. erectus* adaptability across diverse environmental contexts.

**Results:**

Forest plantation populations exhibited impressive shoot growth and moderate root lengths, with plants generally tall and well-weighted. Physiologically, they had moderate chlorophyll content and low carotenoid levels, with a balanced chlorophyll a/b ratio, indicating stable photosynthetic activity. Anatomically, these populations had thicker epidermal and cortical root layers but smaller vascular bundles and phloem regions. Stem and leaf structures were generally moderate in size, with thicker midribs and cortical layers in the leaves. Agricultural field populations showed robust shoot and root systems with balanced fresh and dry biomass. They exhibited high chlorophyll and carotenoid levels, indicating strong photosynthetic capacity. Root and stem anatomy revealed larger root areas, thicker cortex, and wide vascular bundles, reflecting enhanced structural development. Leaves from these populations had moderate midrib and cortical thickness, with larger stomatal areas, promoting efficient gas exchange. Roadside populations displayed deeper roots and reduced biomass production. These populations adapted to environmental stress through leaf expansion, with high leaf numbers and areas. Physiologically, populations had high chlorophyll content, with a high chlorophyll a/b ratio. Root and stem anatomy showed compact structures with smaller vascular bundles, indicating adaptation to harsher conditions. Leaf anatomy was moderate, with smaller vascular bundles and reduced water transport capacity. Wasteland populations exhibited poor growth and high shoot biomass despite small leaves. Physiologically, these populations had the highest total soluble protein and proline contents, reflecting stress adaptation. Anatomically, root and stem structures were variable, with some populations showing reduced cortical cell areas and smaller vascular bundles, indicating limited resource transport. Leaf structures had thicker lamina, thinner epidermal layers, and lower stomatal densities, reflecting adaptation to nutrient-poor soils.

**Conclusion:**

This study reveals the adaptability and thriving potential of *Conocarpus erectus* across varied habitats, providing key insights into its resilience and survival strategies. Understanding these adaptive traits can support habitat restoration, conservation planning, and improve species management in diverse environmental conditions, especially in response to climate change and habitat degradation.

**Supplementary Information:**

The online version contains supplementary material available at 10.1186/s40529-024-00440-0.

## Background

Environmental gradients directly and indirectly shape vegetation dynamics and structure, influencing plant traits and characteristics within ecosystems (Basharat et al. 2024a, b). Ecological dominance of plants is achieved through the adaptive modification of their microstructural and functional traits in response to heterogeneous environmental conditions (Ahmad et al. 2023). These adaptations include changes in leaf anatomy, stomatal density, and cuticular thickness, which enhance water-use efficiency, photosynthesis, and stress tolerance (Ahmad et al. 2022; Irshad et al. 2024). By optimizing these traits, plants can effectively outcompete others in variable environments, maintaining dominance in ecosystems that experience fluctuating climatic and edaphic factors (Wang et al. 2020).

Vegetation classification can be based on physiognomy, functional traits, or species composition, grouping plants with specific structural and environmental tolerances into distinct communities (Boet et al. 2020). These plant associations, which form some of the Earth's largest biomes, reveal critical insights into ecosystem structure, species interactions, and habitat dynamics (Miao et al. 2017; Collins et al. 2020). Both biotic and abiotic factors, including topography and edaphic conditions, strongly affect plant diversity, composition, and distribution (Asanok et al. 2017). Topography influences climate and evapotranspiration, while soil characteristics, shaped by climate, organisms, and parent material, further impact plant growth (Khan and Ahmad 2015). Quantitative analyses are vital for understanding vegetation dynamics and revising their ecological impacts (Mumshad et al. 2021).

Species are often classified by population size, with abundant species recognized as ecologically important. However, terms like "common" or "dominant" vary in usage. Rabinowitz (1981) defined dominant species by large local populations, noting some are widespread ("common"), others have limited habitat specificity ("predictable" or "endemics"). Gaston (2010) and Hanski (1982) describe common species as abundant and widespread, while Mariotte (2014) considers species with > 12% relative abundance as dominant. Despite abundance, some species, termed "subordinates" by Grime (1998), may have minimal ecosystem impact. Highly abundant species are often expected to strongly influence ecological processes, such as food web structure and ecosystem functions (Grime 2006; Gaston 2010). Grime’s mass ratio hypothesis predicts that abundant species, due to their biomass, contribute significantly to energy flow, biogeochemical cycling, and degradation processes. However, not all abundant species have large impacts, those with minimal effects are termed "subordinates". The conflation of dominance and abundance in literature can lead to confusion, hindering accurate predictions. This distinction is vital as anthropogenic changes often reduce dominant species, causing cascading effects on biodiversity and ecosystems (Hillebrand et al. 2008; Gaston 2010).

Widespread species tend to exhibit high intraspecific variation, driven by local adaptation and acclimation across diverse environments (Siefert et al. 2015). This variation allows them to respond flexibly to biotic and abiotic factors, broadening their ecological amplitude (EI-Juhany and Aref 2005). Such variation is key to evolutionary processes, making these species prime candidates for studying speciation (Bolnick et al. 2018). Functional traits—morphological, physiological, and behavioral characteristics—adjust in response to environmental changes and directly impact plant fitness and ecosystem functions (Violle et al. 2007). Instead of responding individually, traits often co-vary in reaction to environmental shifts. However, covariation between traits is rarely considered in studies, potentially leading to misinterpretation of trait dynamics if analyzed in isolation (Pigliucci 2003; Nicotra et al. 2010). Accounting for these interactions is essential for accurate ecological and evolutionary understanding.

*Conocarpus erectus* L. (Combretaceae) is a fast growing, perennial woody and evergreen tree species (EI-Juhany and Aref 2005; Asif et al. 2014). It is widely grown in tropical and sub-tropical regions across the world. It can easily flourish in less fertile soil and tolerate high temperature. Furthermore, it is widely used fodder crop in having high foliar proteins content. Due to fast growing nature, it had invaded the major cities of Pakistan (Al-Humaid 2005; Suleiman et al. 2013). Considering *C. erectus* ability to thrive in diverse ecological settings, it is hypothesized that this species has evolved specific structural and functional adaptations to inhabit a wide range of habitats, and a better understanding of these adaptations can provide insights into its invasive behavior in major cities of Pakistan. The research question addressed in this study were: 1) what structural and functional mechanisms has *C. erectus* developed to cope with environmental stressors such as high temperatures and limited nutrient availability, and how do these mechanisms contribute to its fast growth and invasive nature? ii) What modifications were brought about during the ecological success of *C. erectus*? Hence, it is crucial to understand the underlying mechanism involved in vigorous growth and survival of *C. erectus* under variable environmental conditions. This study on *C. erectus* holds significant future importance in the context of carbon sequestration and ecological stability. Understanding its role in carbon capture and storage can contribute to more effective strategies for mitigating climate change, while its ecological interactions can offer insights into fostering resilient ecosystems and promoting biodiversity conservation. This research is crucial in our efforts to address environmental challenges and enhance the long-term sustainability of our planet.

## Materials and methods

### Study surveys and plant sampling

**Fig. 1:**
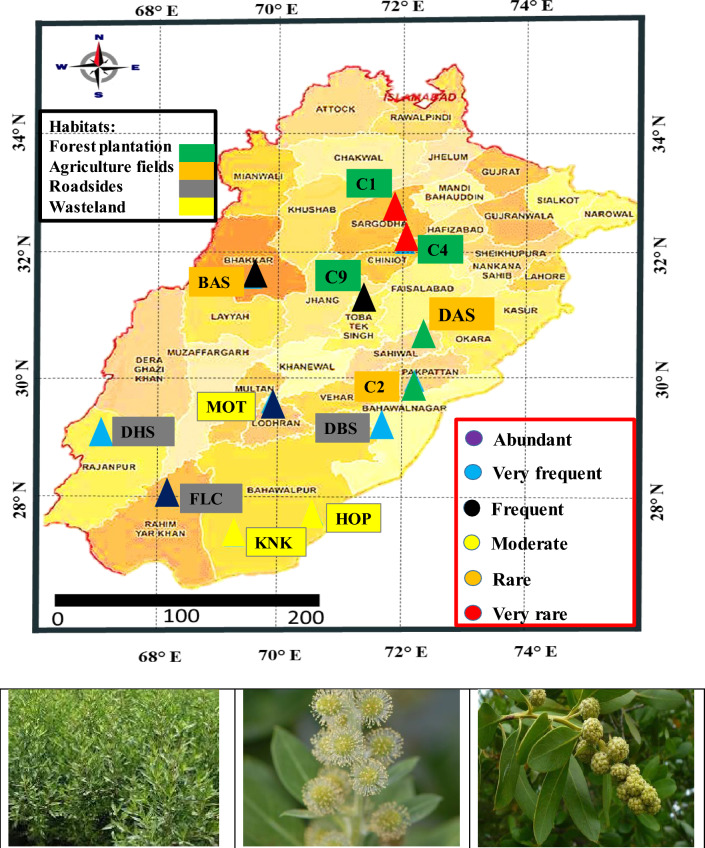
Representing the Punjab map containing collection sites and pectoral view of *Conocarpus erectus* collected from diverse habitats. C1-Chak 1; C4-Chak 4; C9-Chak 9; BAS- Basti Sheikh; C2-Chak 2p; DAS- Dari Sangi; DBS- Dera Bibi Sugra; DHS- Dhoop Sari; FLC- Flood Colony; HOP- Hospital 1p; KNK- Khanpur Nawan Kot; MOT- Model Town

This study focused on *Conocarpus erectus* L. to investigate its adaptability across diverse environments. Twelve populations of *C. erectus* (commonly known as button mangrove or buttonwood) were selected after extensive surveys in Punjab, Pakistan. These populations were located in varied habitats: Chak 1 (artificial forest plantation), Chak 4 (nursery area), Chak 96 (palm area), Basti Sheikh (cotton field), Chak 2p (sugarcane field), Dari Sangi (rice field), Dera Bibi Sugra (roadside), Dhoop Sari (roadside), Flood Colony (roadside), Hospital (disturbed land), Khanpur Nawan Kot (playground), and Model Town (wasteland). Sampling was conducted during peak flowering season with six replicates per site (Fig. 1). The data related to annual temperature and rainfall was taken from Meteorological Department substation situated in each tehsil of district (Table 1, Fig. 1). A GPS system was used to measure the coordinates and elevation of each collection site (Etrex 20 CAN310, Garmin, USA).

**Table 1:** Habitat types, coordinates and meteorological conditions of *Conocarpus erectus* L. populations collected from the Punjab province

Ecological regions	Collection sites	Habitat types	Latitude (N)	Longitude (E)	Elevation (m.a.s.l)	Max. Temp (°C)	Min. Temp (°C)	Rainfall (mm)
Near forest plantation	Chak 1	Artificial forest	28° 66 ′89″	70° 74′ 29″	85	44	13	112
	Chak 4	Nursery area	28° 39′ 31″	70° 33′ 20″	86	42	11	104
	Chak 9	Palm area	31° 28′ 68''	73° 41′ 58''	189	43	10	101
Near agriculture fields	Basti Sheikh	Cotton field	28° 64′ 49″	70° 74′ 14″	90	39	8	123
	Chak 2p	Sugarcane field	*28° 39′ 50"*	*70° 43′ 55"*	91	37	9	131
	Dari Sangi	Rice field	28° 81′ 39″	70° 67′ 67″	314	45	12	117
Near roadsides	Dera Bibi Sugra	Along roadside	28° 25′ 12″	70° 18′ 00″	150	40	10	121
	Dhoop Sari	Near roadside	28° 25′ 12″	70° 18′ 00″	83	38	9	137
	Flood Colony	Roadside	28° 41′ 96″	70° 18 ′09″	82	41	11	115
Along wastelands	Hospital 1p	Barren land	*28*° *42*′ *57*″	70° 29 ′74″	70	39	9	107
	Khanpur Nawan Kot	Near playground	28° 40′ 67″	70° 31′ 94″	90	42	10	127
	Model Town	Wasteland	28° 40′ 98″	70° 30′ 59″	63	43	11	110

### Soil physicochemical parameters

Soil samples were collected at two different depths, 30 and 50 cm to make composite samples for analysis. To determine the ionic contents of the soil, a soil saturation paste was prepared using the collected samples, and a flame photometer (PFP-7, Jenway, UK) was utilized to measure the levels of Ca^2+^, K^+^, and Na^+^. For measuring chloride content, a digital chlorimeter (Model 926, Sherwood Scientific Ltd. Cambridge, UK) was employed. The soil pH and electrical conductivity (ECe) were determined by extracting the soil and using a pH/EC meter (pH/Cond 720, WTW series InoLab, USA). The soil phosphate was measured following the protocol outlined by Wolf (1982). Soil texture was determined using the USDA textural triangle, while organic matter content was analyzed using the method described by Walky (1947). Soil saturation percentage was determined by preparing a saturation paste using 200 g of soil that had been dried in an oven at 70 °C. The formula used to measure the saturation percentage was:

$$SP \left(\%\right)=\frac{Amount \,of \,water\, added\left(g\right)}{Oven\, dried\, soil \left(g\right)} \times100$$

where SP % is saturation percentage.

### Morphological analysis

Three tertiary branches were selected from each plant to measure the several growth parameters. Shoot and root length was measured with the help of scale (Fig. S1). Shoot fresh weight directly measured after sampling but subjected to oven drying at 65 °C to achieve constant weight for measuring shoot dry weight via digital loading balance. Leaf area was measured by using the formula recognized by Lopes et al (2016).

Total leaf area = Maximum leaf length × maximum leaf width × C.F.

C.F. = Correction factor = 0.75.

Overall, the morphological data obtained from tertiary branches was averaged and treated as single replicate.

### Physiological analysis

#### Organic osmolytes

Fully mature sun exposed mature leaves (0.5 g) were taken in falcon tube for analysis of organic osmolytes. Proline was determined after homogenizing the fresh leaf samples in sulfo-salicylic acid which was latterly transferred into ninhydrin solution. These solutions were kept in water bath at 100 °C for one hour and toluene dye was added for final extraction. The samples reading was taken on digital spectrophotometer at 520 nm wavelength.

$$Proline \left(\mu mol{ g}^{-1}fresh\, weight\right)=\frac{\mu \,g\, proline {ml}^{-1}\times\, ml\, of\, toluene/115.5}{ sample \,weight\, (g)}$$

To extract glycine betaine (GB) from the samples, each 0.5 g sample was ground and soaked in 20 ml of deionized water for 24 h at a temperature of 25 °C. The resulting extract was then analyzed using the protocols outlined by Grattan and Grieve (1998). For the determination of total soluble proteins, a fresh leaf sample weighing 0.5 g was sliced and thoroughly ground in 5 ml of phosphate buffer at pH 7.0. The extract was then subjected to centrifugation at 5000 × g for 5 min, and the supernatant was collected for measuring soluble protein levels according to the method described by Lowry et al. (1951).

#### Photosynthetic parameters

A fresh leaf sample of 0.5 g size was grounded in 80% acetone (5 ml) by using a pestle and mortar to achieve their leaf (Arnon 1949; Davis 1979). It was placed in a room for 24 h, and then centrifuged at 1000 rpm for 5 min. Quantity of each pigment was assessed at different wavelength via spectrophotometer. The formulas used to measure the final values:

$$Chl. a \left(mg {g}^{-1} f.wt.\right)={\left[12.7\left(\text{OD}663\right)- 2.69\left(\text{OD}645\right)\right]\text{x}\frac{\text{V}}{1000}\text{x W}]}$$

$$Chl. b \left(mg {g}^{-1} f.wt.\right)={\left[22.9\left(\text{OD}645\right)- 4.68\left(\text{OD}663\right)\right]\text{x}\frac{\text{V}}{1000}\text{x W}]}$$

$$Total\, chl. \left(mg {g}^{-1} f.wt.\right)={\left[20.2\left(\text{OD}645\right)- 8.02 \,\left(\text{OD}663\right)\right]\times \frac{\text{V}}{1000}\times\text{W}]}$$

$$Carotenoids \left(mg {g}^{-1} f.wt.\right)={\left[12.7\left(OD480\right)- 0.114 \left(OD663\right)\right]-0.638 (OD645)]/2500}$$

#### Anatomical analysis

The different plant parts were sampled from each tree branch for anatomical findings. A 2-cm piece from lateral roots, topmost internode of shoot and fully mature leaf was taken for anatomical sections. These were passed through formalin acetic alcohol for fixation, and acetic alcohol for long term preservation of samples (Ahmad et al. 2023; Basharat et al. 2024a, b). Sectioning as well as staining of each sample was done for permanent slide preparation. For staining, safranin and fast green were used to improve the tissues contrasts and differentiation (Ruzin 1999). A dissecting microscope with digital camera was used to take photographs for seeing difference in tissues systems (Ahmad et al. 2022).

### Statistical analysis

To address the research questions such as i) which attributes (structural and functional) are contributing to ecological success of *C. erectus* was addressed by using one-way analysis of variance (ANOVA). Least significance difference (LSD) was calculated, and their values were used for comparison of mean values of observed traits (Zafar et al. 2024), by using Costate statistical package (CoStat v. 6.303, Monterey, CA, USA), and ii) whether the heterogeneity in habitat types and soil factors determine the propagation and survival of species was tackled by constructing clustered heatmaps among soil physicochemical features and species variables by using R statistical software (R Development Core Team 2017). Principal component analysis (PCA) was conducted in R software (version 4.1.1) to assess the relationships between structural and functional traits and soil factors across different habitats (Shehzad et al. 2023).

## Results

### Soil physicochemical characteristics

Forest plantations habitats like Chak 1 exhibited sandy soils with low salinity and moderate pH levels (Table 2). Potassium (K^+^), sodium (Na^+^), and calcium (Ca^2+^) concentrations were generally lower than other habitats, while organic matter (OM) content was higher. The soils here had a good saturation percentage (SP), suggesting moderate moisture retention. Agricultural fields, such as Chak 4 and Chak 9, had loamy soils with higher salinity (ECe) and slightly alkaline pH values. Potassium and sodium levels were significantly higher in these fields. The organic matter content was moderate, and the soils exhibited a good amount of phosphorus (PO_4_). Roadside habitats, such as Dera Bibi Sugra and Dhoop Sari, displayed varying soil textures, with loamy or loamy sand soils. Salinity levels were moderate to high, and the pH values were slightly alkaline. Potassium and sodium concentrations were moderate, while organic matter content was lower than in other habitats. These soils generally had moderate levels of calcium and chloride, with sufficient phosphorus content. Wasteland sites, such as Flood Colony and Khanpur Nawan Kot, presented extreme soil conditions. For instance, Flood Colony had the highest salinity and sodium content, along with high levels of calcium and chloride, indicating soil salinization. The organic matter content was very low, and phosphorus availability was moderate. In contrast, other wasteland sites, like Khanpur Nawan Kot, had sandy soils with relatively lower nutrient content but a higher saturation percentage.

**Table 2:** Soil physicochemical parameters of *Conocarpus erectus* L. populations collected from the Punjab province

Collection sites	Soil texture	ECe (dS/m)	pH	K^+^ (mg K^−1^)	Na^+^ (mg K^−1^)	Ca^2+^ (mg K^−1^)	Clˉ (mg K^−1^)	SP (%)	OM (%)	PO_4_ (ppm)
Chak 1	Sandy	0.45f ± 0.3	7.2d ± 0.1	60i ± 5.5	81.1 g ± 3.5	72.6f ± 4.5	125.6 g ± 5.3	27c ± 2.4	0.53a ± 0.05	2.1d ± 0.1
Chak 4	Loamy	3.67de ± 0.2	8.4ab ± 0.1	65 h ± 5.3	314.3e ± 5.6	40.5i ± 3.7	197.4e ± 5.3	18e ± 1.4	0.24d ± 0.03	4.6a ± 0.2
Chak 9	Loamy	5.12b ± 0.3	8.3ab ± 0.1	250c ± 10.1	524.1c ± 10.3	55.8 h ± 4.8	232.8c ± 5.7	23d ± 1.6	0.43b ± 0.05	3.4bc ± 0.2
Basti Sheikh	Sandy	2.11e ± 0.1	7.7c ± 0.1	60 h ± 5.3	194.2 ± 5.6	35.3j ± 3.5	103.5 h ± 5.8	35a ± 2.5	0.34c ± 0.03	1.8e ± 0.1
Chak 2p	Loamy	4.36c ± 0.2	8.0b ± 0.1	150f ± 10.2	433.7d ± 10.1	89.9e ± 4.4	17.7 k ± 2.4	16f ± 1.2	0.51a ± 0.05	1.2f ± 0.1
Dari Sangi	Sandy	0.61 g ± 0.03	7.6c ± 0.1	160e ± 5.8	64.2 h ± 2.3	65.7 g ± 3.4	42.1j ± 2.2	13 g ± 1.2	0.24d ± 0.03	2.9c ± 0.1
Dera Bibi Sugra	Loamy sand	3.43d ± 0.2	8.8a ± 0.1	286b ± 10.3	311.5e ± 5.7	58.1 h ± 3.5	155.2f ± 5.6	25c ± 1.5	0.45b ± 0.05	3.2bc ± 0.2
Dhoop Sari	Loamy	5.11b ± 0.3	8.7a ± 0.1	134 g ± 5.4	634.3b ± 10.7	326.9c ± 5.2	345.9b ± 5.3	33ab ± 2.2	0.32c ± 0.03	2.1d ± 0.1
Flood Colony	Sandy	6.81a ± 0.4	6.3e ± 0.1	298a ± 10.5	788.5a ± 15.9	477.2a ± 5.7	455.9a ± 10.8	20de ± 2.3	0.11e ± 0.02	3.8b ± 0.2
Hospital 1p	Loamy	5.66ab ± 0.3	7.2d ± 0.1	180d ± 10.2	634.3b ± 10.4	356.9b ± 5.8	345.6b ± 5.6	22d ± 1.5	0.33c ± 0.03	1.2f ± 0.1
Khanpur Nawan Kot	Sandy	1.23f ± 0.1	8.4ab ± 0.1	55j ± 5.5	98.2f ± 3.7	32.6 k ± 2.8	56.2i ± 2.8	16f ± 1.1	0.54a ± 0.05	2.9c ± 0.1
Model Town	Loamy sand	3.12d ± 0.2	7.7c ± 0.1	156f ± 10.7	311.9e ± 5.8	116.4d ± 4.9	211.0d ± 5.9	10 h ± 1.2	0.33c ± 0.03	3.3bc ± 0.2
	**LSD**	0.7	1.0	10.5	24.9	37.4	43.9	4.5	0.1	0.5

Means followed by same letters in each column are not significant at p ≤ 0.05

### Morphological characteristics

Populations located near forest plantations, such as C1, displayed impressive shoot growth, with the highest shoot length observed (Table 3, P < 0.05). These populations also had moderate to high root lengths, fresh shoot weight, and dry shoot weight. This habitat generally supports the development of tall and well-weighted plants. Additionally, leaf numbers were average, but leaf areas were smaller compared to other habitats. Populations near agricultural fields, including C2 and DAS, exhibited considerable shoot lengths and robust root systems. The plants from these sites showed balanced fresh and dry shoot weights, while their leaf numbers per branch were high. The leaf area was among the largest across all populations. Populations growing along roadsides, such as C4 and C9, showed mixed characteristics. While C9 had the longest roots, indicating a deep-rooting ability possibly to access water in harsh conditions, C4 had much shorter roots and the lowest shoot dry weight. Leaf number and leaf area were both relatively high in C4, suggesting that roadside populations adapt through leaf expansion. Wasteland populations, including BAS, DHS, KNK, and MOT, displayed a wide range of traits, often reflecting the challenging conditions of these habitats. BAS had the shortest shoot length, lowest leaf numbers, and small leaf area, reflecting poor growth conditions. Conversely, MOT showed the highest shoot fresh and dry weights despite having the smallest leaves, indicating that some populations in wastelands might invest more in biomass accumulation than leaf expansion. KNK had minimal root weight, suggesting poor root development in degraded soils.

**Table 3:** Morphological and physiological characteristics of *Conocarpus erectus* L. populations collected from the Punjab province

Habitat types	Near forest plantation			Near agriculture fields			Along roadsides			Along wastelands				
Collection sites	C1	C4	C9	BAS	C2	DAS	DBS	DHS	FLC	HOP	KNK	MOT	LSD	F-ratio
*Morphological characteristics*														
Shoot length (cm)	37.0c ± 0.7	30.0e ± 0.5	26.0f ± 0.6	19.0 g ± 0.5	53.0a ± 0.8	38.0c ± 0.6	38.0c ± 0.6	30.0e ± 0.5	38.0c ± 0.7	46.0b ± 0.5	47.0b ± 0.5	35.0d ± 0.7	8.9	12.5**
Root length (cm)	12.5e ± 0.4	9.0f ± 0.3	38.0a ± 0.5	27.0b ± 0.4	13.0e ± 0.3	16.0d ± 0.5	18.0c ± 0.5	20.0c ± 0.5	13.0e ± 0.4	9.0f ± 0.3	13.0e ± 0.4	25.0b ± 0.5	12.3	8.9**
Shoot fresh weight (g plant⁻^1^)	12.0c ± 0.5	15.0b ± 0.4	15.0b ± 0.4	12.0c ± 0.5	11.5c ± 0.4	16.8b ± 0.5	8.7d ± 0.5	15.3b ± 0.4	16.0b ± 0.5	19.5a ± 0.6	14.0bc ± 0.5	20.0a ± 0.5	5.5	5.3*
Shoot dry weight (g plant⁻^1^)	4.0c ± 0.2	2.7e ± 0.1	4.7c ± 0.2	3.3d ± 0.2	4.0c ± 0.2	5.6b ± 0.2	3.3d ± 0.2	5.1b ± 0.2	4.0c ± 0.2	6.5ab ± 0.2	3.5d ± 0.1	7.5a ± 0.2	1.5	2.8*
Root fresh weight (g plant⁻^1^)	2.3d ± 0.2	2.1d ± 0.1	2.0d ± 0.1	4.0bc ± 0.3	1.6e ± 0.2	2.1d ± 0.1	2.4d ± 0.2	4.5b ± 0.2	8.5a ± 0.3	1.0f ± 0.1	1.0f ± 0.1	3.0c ± 0.2	2.0	1.4^ ns^
Root dry weight (g plant⁻^1^)	1.0b ± 0.1	0.3e ± 0.1	0.4de ± 0.1	1.3b ± 0.1	0.5d ± 0.1	0.7c ± 0.1	0.8c ± 0.1	1.5b ± 0.1	2.4a ± 0.1	0.3e ± 0.1	0.1f ± 0.1	0.9c ± 0.1	1.0	1.0^ ns^
Leaf number per branch	31.0e ± 0.5	62.0b ± 0.5	28.0e ± 0.5	17.0f ± 0.5	53.0c ± 0.5	27.0e ± 0.5	25.0ef ± 0.5	40.0d ± 0.5	60.0b ± 0.5	74.0a ± 0.5	30.0e ± 0.5	67.0b ± 0.5	12.1	23.5**
Leaf area (cm^2^)	19.4c ± 0.5	27.3a ± 0.5	24.4b ± 0.5	11.8d ± 0.5	18.5c ± 0.5	26.6a ± 0.5	22.6b ± 0.5	13.8d ± 0.5	24.4b ± 0.5	26.6a ± 0.5	19.7c ± 0.5	9.8e ± 0.5	2.1	9.7**
*Physiological characteristics*														
Total soluble proteins (μg g⁻^1^ f.wt.)	224.7i ± 5.0	107.8 k ± 3.0	585.0d ± 10.0	342.5 g ± 7.0	381.7f ± 8.0	152.1j ± 4.0	326.9 h ± 6.0	453.3e ± 8.0	660.1c ± 12.0	452.9e ± 8.0	727.5b ± 12.0	749.7a ± 10.0	53.4	57.3***
Proline (μmol g⁻^1^ f.wt.)	38.7f ± 1.5	54.3c ± 1.2	44.6e ± 1.3	50.5c ± 1.3	42.9e ± 1.2	47.6d ± 1.2	46.9d ± 1.2	19.3 g ± 0.5	66.0b ± 1.5	61.3b ± 1.5	73.5a ± 1.5	39.7f ± 1.2	12.0	21.4**
Glycine betaine (μmol g⁻^1^ f.wt.)	18.2c ± 0.5	18.2c ± 0.5	14.4d ± 0.5	30.8a ± 0.8	12.1de ± 0.3	5.3f ± 0.2	17.2c ± 0.5	5.3f ± 0.2	13.8de ± 0.4	21.4bc ± 0.6	22.2b ± 0.5	18.4c ± 0.5	8.9	14.7**
Chlorophyll a (mg g⁻^1^ f.wt.)	1.2d ± 0.1	1.5c ± 0.1	1.1d ± 0.1	1.2d ± 0.1	2.0b ± 0.1	2.0b ± 0.1	2.1b ± 0.1	2.5a ± 0.1	2.1b ± 0.1	1.6c ± 0.1	0.8e ± 0.1	1.0d ± 0.1	1.5	1.4**
Chlorophyll b (mg g⁻^1^ f.wt.)	0.8d ± 0.1	1.3b ± 0.1	1.1c ± 0.1	1.9a ± 0.1	0.8d ± 0.1	1.4b ± 0.1	0.5e ± 0.1	0.8d ± 0.1	0.5e ± 0.1	0.4f ± 0.1	0.3 g ± 0.1	0.8d ± 0.1	1.0	0.3**
Total Chlorophyll (mg g⁻^1^ f.wt.)	2.0f ± 0.2	3.3a ± 0.2	2.2e ± 0.2	3.1b ± 0.2	2.8c ± 0.2	3.4a ± 0.2	2.6d ± 0.2	3.3a ± 0.2	2.6d ± 0.2	2.0f ± 0.2	1.1 h ± 0.1	1.8 g ± 0.2	2.0	2.0**
Carotenoids (mg g⁻^1^ f.wt.)	0.3c ± 0.05	0.4b ± 0.05	0.3c ± 0.05	0.8a ± 0.1	0.3c ± 0.05	0.2d ± 0.05	0.1e ± 0.01	0.1e ± 0.01	0.1e ± 0.01	0.2d ± 0.05	0.1e ± 0.01	0.2d ± 0.05	0.1	0.5**
Chlorophyll a/b	1.5d ± 0.1	1.1e ± 0.05	1.0e ± 0.05	0.6f ± 0.1	2.5c ± 0.1	1.4d ± 0.1	4.2a ± 0.1	3.1b ± 0.1	4.2a ± 0.1	4.0a ± 0.1	2.7c ± 0.05	1.3d ± 0.1	1.0	1.5**
Chlorophyll /Carotenoids	6.7f ± 0.5	8.5e ± 0.5	7.3ef ± 0.5	3.9 g ± 0.5	9.3d ± 0.5	17.0c ± 0.5	26.0b ± 0.5	33.0a ± 0.5	26.0b ± 0.5	10.0d ± 0.5	11.0d ± 0.5	9.0d ± 0.5	7.0	3.1**

Means followed by same letters in each row are not significant at p ≤ 0.05

^*^, significant at P < 0.05; **, significant at P < 0.01; ***, significant at P < 0.001; NS, not significant

### Physiological characteristics

Populations near forest plantations (C4) exhibited low total soluble protein content (Table 3, P < 0.05). Chlorophyll contents (a and b) was moderate, while carotenoid levels were relatively low. These populations showed a balanced chlorophyll a/b ratio, indicating stable physiological activity under this habitat. Populations from agricultural fields (BAS) had high glycine betaine content, indicating notable physiological responses. These populations also showed high levels of chlorophyll b and total chlorophyll, with carotenoid levels being the highest among all habitats. However, the chlorophyll/carotenoid ratio was lower compared to other habitats. Roadside populations, including DHS and FLC, exhibited high chlorophyll a and total chlorophyll content. Carotenoid levels were low, while the chlorophyll a/b ratio was high, particularly in DHS and FLC. These populations showed higher chlorophyll/carotenoid ratios compared to other habitats. Wasteland populations, such as MOT and KNK, displayed the highest total soluble protein content, especially in MOT. Proline content was highest in KNK, while this population also had the lowest chlorophyll levels (a, b). Carotenoid levels were minimal in KNK, and the chlorophyll a/b ratio was notably high in some wasteland populations like HOP. The F-ratio of soluble proteins (57.3***), at p < 0.001, proline (21.4**) and glycine betaine (17.7**) at p < 0.01 showed significant different *Conocarpus erectus* populations in terms of physiological attributes. All populations of *C. erectus* exhibited significant variation in photosynthetic traits (F-ratio) across diverse habitats.

### Anatomical characteristics

All the populations of *C. erectus* showed significant variations related to anatomical traits such as dermal tissues (epidermis and cuticle), mechanical tissues (collenchyma and sclerenchyma), and vascular tissues (metaxylem vessels and phloem region and storage tissues (cortex and pith) in response to variable environmental condition. All populations of *C. erectus* exhibited highly significant variation in anatomical traits (F-ratio) across diverse habitats for root area (211.3***), vascular bundle area (35.2***), stem area (89.4***), pith thickness (76.3***), midrib thickness (115.7***) and stomatal number (58.4***) as depicted in Table 4.

**Table 4:** Anatomical characteristics of *Conocarpus erectus* L. populations collected from the Punjab province

Habitat types	Near forest plantation			Near agriculture field			Along roadside			Along wasteland				
Collection sites	C1	C4	C9	BAS	C2	DAS	DBS	DHS	FLC	HOP	KNK	MOT	LSD	F-ratio
*Root anatomy*														
Root area (µm^2^)	367.4f ± 5.0	408.0b ± 6.0	320.3 g ± 4.0	372.1e ± 5.0	395.6c ± 5.0	447.5a ± 7.0	409.8b ± 6.0	226.1i ± 3.0	362.7f ± 5.0	235.5 h ± 4.0	381.5d ± 5.0	386.2d ± 5.0	37.6	211.3***
Epidermal thickness (µm)	23.6a ± 0.5	9.4d ± 0.3	9.4d ± 0.3	18.8b ± 0.4	23.6a ± 0.5	14.1c ± 0.3	9.4d ± 0.3	4.7e ± 0.2	18.8b ± 0.4	14.1c ± 0.3	18.8b ± 0.4	14.1c ± 0.3	5.4	5.4**
Cortical thickness (µm)	84.8c ± 1.0	131.9a ± 2.0	23.6 h ± 0.5	75.4d ± 1.0	122.5b ± 1.5	131.9a ± 2.0	42.4f ± 0.8	23.6 h ± 0.5	47.1f ± 0.8	42.4f ± 0.8	33.0 g ± 0.6	65.9e ± 1.0	9.8	23.7**
Cortical cell area (µm^2^)	14.1c ± 0.5	18.8b ± 0.5	9.4d ± 0.3	4.7e ± 0.2	14.1c ± 0.5	23.6a ± 0.5	14.1c ± 0.5	9.4d ± 0.3	9.4d ± 0.3	14.1c ± 0.5	4.7e ± 0.2	9.4d ± 0.3	4.9	3.1^ ns^
Vascular bundle area (µm^2^)	65.9f ± 1.0	75.4e ± 1.0	94.2c ± 1.0	108.3b ± 1.0	47.1 g ± 0.8	61.2f ± 0.8	113.0a ± 2.0	56.5 g ± 0.8	84.8d ± 1.0	37.7 h ± 0.5	61.2f ± 0.8	94.2c ± 1.0	5.0	35.2***
Metaxylem area (µm^2^)	9.4c ± 0.2	4.7d ± 0.2	18.8a ± 0.5	14.1b ± 0.4	14.1b ± 0.4	4.7d ± 0.2	9.4c ± 0.3	9.4c ± 0.3	9.4c ± 0.3	4.7d ± 0.2	9.4c ± 0.3	9.4c ± 0.3	3.8	2.2^ ns^
Phloem area (µm^2^)	33.0d ± 0.5	37.7c ± 0.6	18.8 g ± 0.4	42.4b ± 0.5	18.8 g ± 0.4	14.1 h ± 0.3	33.0d ± 0.5	28.3e ± 0.5	33.0d ± 0.5	23.6f ± 0.4	42.4b ± 0.5	51.8a ± 0.8	6.9	13.3**
*Stem anatomy*														
Stem area (µm^2^)	471.0c ± 5.0	428.6f ± 4.0	471.0c ± 5.0	348.5 g ± 3.5	449.3e ± 4.5	400.4 h ± 4.0	541.7ab ± 6.0	423.9f ± 4.0	325.0i ± 3.0	546.4a ± 6.0	508.7b ± 5.0	456.9d ± 4.0	41.5	89.4***
Epidermal thickness (µm)	18.8b ± 0.5	14.1c ± 0.3	4.7e ± 0.2	9.4d ± 0.3	9.4d ± 0.3	18.8b ± 0.5	23.6a ± 0.5	14.1c ± 0.3	9.4d ± 0.3	14.1c ± 0.3	14.1c ± 0.3	9.4d ± 0.3	6.7	11.7**
Cortical thickness (µm)	61.2c ± 1.0	70.7b ± 1.0	23.6 g ± 0.5	28.3f ± 0.5	65.9c ± 1.0	37.7e ± 0.8	37.7e ± 0.8	51.8d ± 1.0	84.8a ± 1.5	28.3f ± 0.5	37.7e ± 0.8	61.2c ± 1.0	14.0	36.5**
Cortical cell area (µm^2^)	9.4d ± 0.3	4.7e ± 0.2	23.6a ± 0.5	4.7e ± 0.2	4.7e ± 0.2	9.4d ± 0.3	18.8b ± 0.5	14.1c ± 0.3	9.4d ± 0.3	4.7e ± 0.2	4.7e ± 0.2	4.7e ± 0.2	7.0	2.1^ ns^
Vascular bundle area (µm^2^)	47.1 h ± 1.0	65.9f ± 1.0	89.5c ± 1.0	70.7e ± 1.0	117.8a ± 2.0	61.2f ± 0.8	47.1 h ± 1.0	75.4d ± 1.0	56.5 g ± 1.0	108.3b ± 1.5	65.9f ± 1.0	61.2f ± 0.8	8.9	45.8**
Metaxylem area (µm^2^)	4.7d ± 0.2	4.7d ± 0.2	9.4c ± 0.3	9.4c ± 0.3	9.4c ± 0.3	4.7d ± 0.2	9.4c ± 0.3	14.1b ± 0.5	9.4c ± 0.3	18.8a ± 0.4	4.7d ± 0.2	4.7d ± 0.2	3.5	3.4**
Phloem area (µm^2^)	18.8c ± 0.5	23.6b ± 0.5	23.6b ± 0.5	33.0a ± 0.5	23.6b ± 0.5	23.6b ± 0.5	33.0a ± 0.5	23.6b ± 0.5	14.1d ± 0.3	23.6b ± 0.5	18.8c ± 0.5	14.1d ± 0.3	9.5	17.6**
Pith thickness (µm)	183.7 h ± 2.0	240.2e ± 3.0	259.1d ± 3.0	155.4i ± 2.0	282.6c ± 3.0	150.7j ± 2.0	235.5f ± 3.0	197.8 g ± 2.5	150.7j ± 2.0	296.7b ± 3.0	306.2a ± 3.0	244.9e ± 2.5	10.2	76.3***
Pith cell area (µm^2^)	9.4d ± 0.3	14.1c ± 0.3	18.8b ± 0.4	14.1c ± 0.3	28.3a ± 0.5	14.1c ± 0.3	18.8b ± 0.4	18.8b ± 0.4	14.1c ± 0.3	28.3a ± 0.5	18.8b ± 0.4	9.4d ± 0.3	13.9	12.7**
*Leaf anatomy*														
Midrib thickness (µm)	423.9a ± 5.0	358.0d ± 4.0	372.1c ± 4.0	254.3 h ± 3.0	372.1c ± 4.0	409.8b ± 5.0	376.8c ± 4.0	409.4b ± 5.0	282.6 g ± 3.0	334.4e ± 4.0	334.4e ± 4.0	329.7f ± 4.0	14.0	115.7***
Lamina thickness (µm)	89.5d ± 1.0	89.5d ± 1.0	103.6c ± 1.0	70.7f ± 0.8	80.1e ± 1.0	98.9 cd ± 1.0	94.2 cd ± 1.0	75.4e ± 0.8	127.2b ± 1.5	70.7f ± 0.8	127.2b ± 1.5	141.3a ± 1.5	13.4	34.5**
Epidermal thickness (µm)	18.8b ± 0.5	18.8b ± 0.5	9.4d ± 0.2	23.6a ± 0.5	14.1c ± 0.3	14.1c ± 0.3	18.8b ± 0.5	23.6a ± 0.5	23.6a ± 0.5	14.1c ± 0.3	18.8b ± 0.5	14.1c ± 0.3	4.8	10.9**
Cortical thickness (µm)	188.4a ± 2.0	146.0 cd ± 1.5	150.7c ± 1.5	94.2 g ± 1.0	164.9b ± 2.0	164.9b ± 2.0	150.7c ± 1.5	183.7b ± 2.0	141.3d ± 1.0	127.2f ± 1.0	122.5f ± 1.0	136.6e ± 1.5	24.3	12.3**
Cortical cell area (µm^2^)	18.8a ± 0.5	9.4c ± 0.3	9.4c ± 0.3	18.8a ± 0.5	18.8a ± 0.5	9.4c ± 0.3	14.1b ± 0.4	14.1b ± 0.4	9.4c ± 0.3	9.4c ± 0.3	4.7d ± 0.2	9.4c ± 0.3	3.8	2.7**
Vascular bundle area (µm^2^)	75.4e ± 1.0	70.7e ± 1.0	80.1d ± 1.0	80.1d ± 1.0	75.4e ± 1.0	108.3a ± 2.0	56.5 g ± 1.0	94.2b ± 1.0	65.9f ± 1.0	89.5b ± 1.0	84.8c ± 1.0	65.9f ± 1.0	29.4	58.9**
Metaxylem area (µm^2^)	4.7b ± 0.2	4.7b ± 0.2	4.7b ± 0.2	9.4a ± 0.3	4.7b ± 0.2	9.4a ± 0.3	4.7b ± 0.2	9.4a ± 0.3	9.4a ± 0.3	4.7b ± 0.2	4.7b ± 0.2	9.4a ± 0.3	4.8	1.5^ ns^
Phloem area (µm^2^)	23.6b ± 0.5	14.1d ± 0.3	28.3a ± 0.5	28.3a ± 0.5	28.3a ± 0.5	18.8c ± 0.4	14.1d ± 0.3	23.6b ± 0.5	9.4e ± 0.3	9.4e ± 0.3	9.4e ± 0.3	23.6b ± 0.5	5.6	6.3**
Stomatal number (/mm^2^)	329.0a ± 5.0	249.3d ± 4.0	268.7c ± 4.0	211.5e ± 3.0	249.3d ± 4.0	169.6f ± 4.0	324.9ab ± 5.0	327.0a ± 5.0	267.6c ± 4.0	205.3e ± 3.0	168.5f ± 4.0	286.0b ± 4.0	4.5	58.4***
Stomatal area (µm^2^)	18.8a ± 0.5	18.8a ± 0.5	14.1b ± 0.3	14.1b ± 0.3	14.1b ± 0.3	18.8a ± 0.5	4.7d ± 0.2	18.8a ± 0.5	9.4c ± 0.3	9.4c ± 0.3	14.1b ± 0.3	14.1b ± 0.3	3.0	3.1^ ns^

Means followed by same letters in each row are not significant at p ≤ 0.05

^*^, significant at P < 0.05; **, significant at P < 0.01; ***, significant at P < 0.001; NS, not significant

#### Root anatomy

**Fig. 2:**
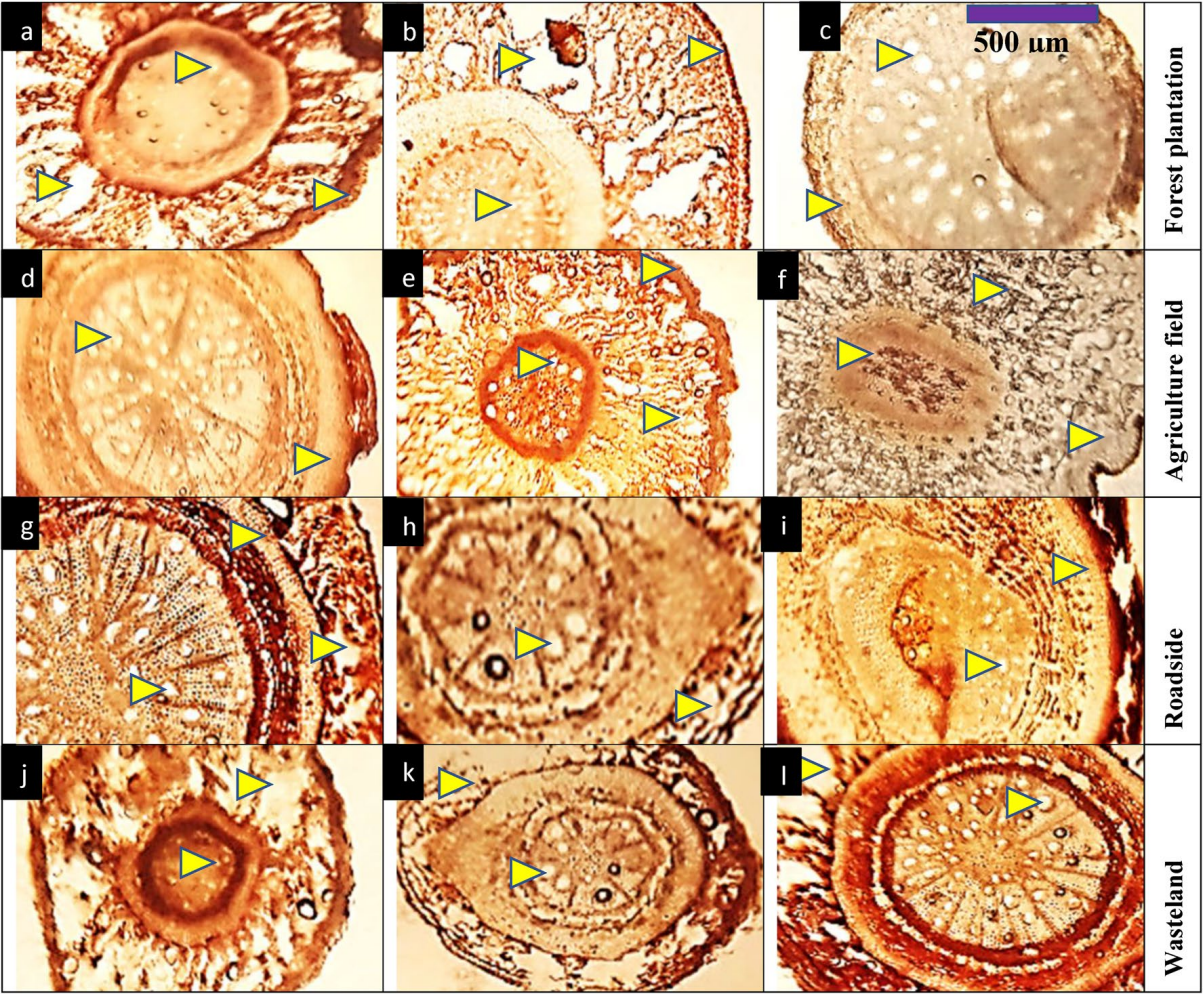
Root transvers sections of *Conocarpus erectus* populations collected from the Punjab province (Mx = 40X; n = 6). Description: 1) forest plantation; **a** Chak 1-thicker epidermis, large lysigenous cavities, reduced metaxylem vessels and pith region, **b** Chak 4-thicker epidermis, partially crushed root region, very reduced metaxylem vessels, **c** Chak 96-reduced root area and partially crushed from one side, enlarge metaxylem vessels. 2) agriculture fields; **d** Basti Sheikh-rounded and thicker root section, large metaxylem vessels and pith region, **e** Chak 2P-thicker epidermis, partially crushed cortical region, reduced metaxylem vessels, **f** Dari Sanjhi-enlarge root cellular area, compact cortical region, very reduced xylem vessels.3) roadsides; **g** Dera Bibi Sughra-partially crushed root cortical region, thick endodermis, enlarge pith region and xylem vessels, **h** Dhoop Sari-very reduced root cellular area and little crushed, greatly reduced metaxylem vessels and pith area, **i** Flood colony-thick epidermis, large cortical region, xylem vessels large and sparsely arranged.4) wastelands; **j** Hospital-greatly reduced root region, large laygenous cavities, reduced pith and metxylem area, **k** Khanpur Nawan kot-thick root and partially crushed, large metaxylem vessels and pith region, **l** Model Town-enlarge root cellular area with large lysigenous cavities, enlarge metaxylem vessels and pith region

Forest plantation populations like C1 and C4 exhibited thicker epidermis and moderate cortical thickness (Table 4, Fig. 2). Root area was moderately large, and vascular bundle and phloem areas were relatively smaller compared to other habitats. Interestingly, C9 had the widest metaxylem area. Agriculture field populations, such as BAS, C2, and DAS, exhibited significantly larger root areas, with DAS recording the highest value. The epidermis was generally thick, particularly in C2 and BAS, indicating robust root structures. Cortical thickness and cell areas were also the largest in these populations, with DAS showing the thickest cortex and the largest cortical cells. This habitat also had the widest vascular bundles and phloem regions, particularly in DBS and MOT. Roadside populations, such as DHS, DBS, and FLC, showed smaller and thinner root structures compared to other habitats. DHS had the smallest root area and the thinnest epidermis, along with reduced cortical thickness and cell area, suggesting that these roots are more compact. The vascular bundle areas were smaller as well, except for DBS, which had a relatively larger vascular bundle area. Phloem areas in this habitat were moderate, though smaller compared to agricultural populations. Wasteland populations, including HOP, KNK, and MOT, showed variable root anatomical features. MOT had a relatively large root area and phloem region. However, HOP exhibited the smallest vascular bundle area, and KNK had reduced cortical cell areas. Despite these challenges, phloem areas were enhanced in MOT, suggesting some adaptation to nutrient limitations in wasteland soils.

#### Stem anatomy

**Fig. 3:**
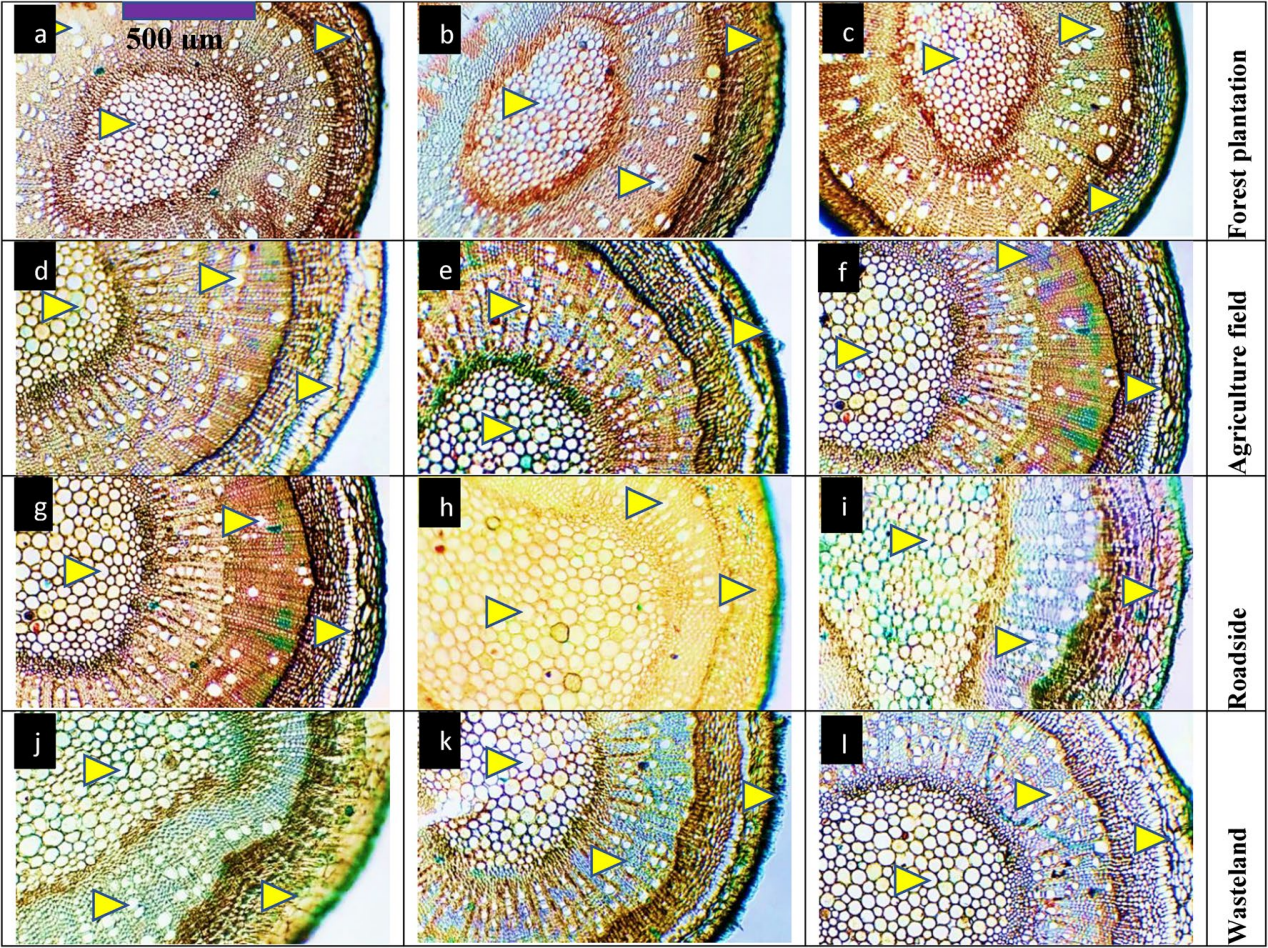
Stem transvers sections of *Conocarpus erectus* populations collected from the Punjab province (Mx = 40X; n = 6). Description: 1) forest plantation; **a** Chak 1-triangular stem, thick epidermis and cortical region, prominent pith region, **b** Chak 4-reduced stem area and cortical region, enhanced pith thickness and metaxylem area, **c** Chak 96-thick stem area, reduced cortical region, enlarged pith region and its cell area. 2) agriculture fields; **d** Basti Sheikh-thick and rounded stem, enlarged pith area, reduced xylem vessels, **e** Chak 2P-enlarged stem cellular area, metaxylem vessels and pith region, **f** Dari Sanjhi-triangular stem, thick cortical region and pith area, enlarged xylem vessels. 3) roadsides; **g** Dera Bibi Sughra-extraordinary thick stem cellular area and epidermis, reduced xylem vessels, **h** Dhoop Sari-triangular stem, enlarged metaxylem vessels and pith region, **i** Flood colony-reduced stem area, cortical region and pith cellular area, enlarged xylem vessels.4) wasteland; **j** Hospital-extraordinary thick stem area, enlarge xylem vessels and pith cells area, **k** Khanpur Nawan kot-thick stem with reduced cortical region and metaxylem area, enlarged pith, **l** Model Town-enlarged stem area with large lysigenous cavities, xylem vessels and pith region

Populations from forest plantation habitats, such as C1, C4, and C9, generally displayed moderate to smaller stem areas and vascular bundles (Table 4, Fig. 3). C9 showed a larger cortical thickness and the largest cortical cells, while stem areas and epidermal thickness were lower, especially in C9 and C1. Agriculture field populations like C2, DAS, and DBS exhibited larger stem areas, with DBS showing the thickest epidermis. C2 had the largest vascular bundle area, while DAS had a more developed stem structure, and DBS showed thicker cortical regions. Roadside populations, including FLC, DBS, and KNK, displayed smaller stem areas overall, particularly in FLC. The cortical thickness varied, with KNK showing a larger pith thickness, while FLC had a thinner pith and smaller vascular bundles. Wasteland populations, such as HOP and MOT, showed variability, with HOP having the largest stem area and widened metaxylem vessels. MOT exhibited a smaller stem area and thinner vascular bundles, with reduced pith size compared to other wasteland populations.

#### Leaf anatomy

**Fig. 4:**
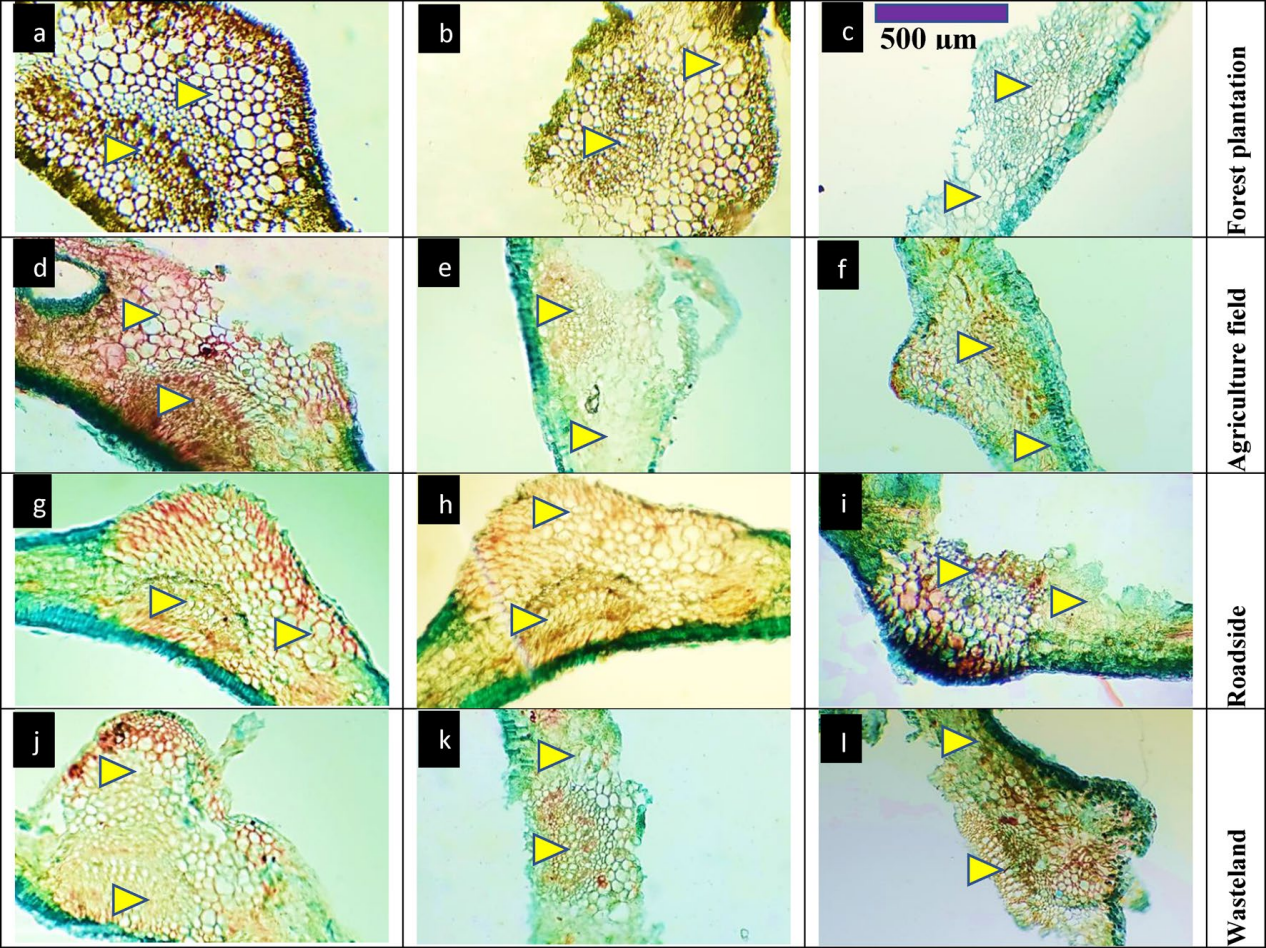
Leaf transvers sections of *Conocarpus erectus* populations collected from the Punjab province (Mx = 40X; n = 6). Description: 1) forest planation; **a** Chak 1- thick leaf with thick lamina and midrib, enlarged cortical parenchyma, reduced vascular bundles, **b** Chak 4-reduced leaf thickness, cortical parenchyma cells large, enlarge vascular bundles, **c** Chak 96-thick leaf with reduced lamina, enlarge cortical parenchyma and vascular region. 2) agriculture fileds; **d** Basti Sheikh-thick leaf with reduced lamina, large cortical parenchyma, narrower vascular bundles, **e** Chak 2P-thick leaf with reduced lamina, enlarged cortical region and vascular bundles area, **f** Dari Sanjhi-thick midrib with reduced lamina having large cortical region, greatly enlarged vascular bundles.3) roadsides; **g** Dera Bibi Sughra-extraordinary thick leaves, enlarged vascular bundles, reduced lamina thickness, **h** Dhoop Sari-elliptical leaves with reduced lamina, enlarged cortical region and vascular bundles area, **i** Flood colony-very thick leaf with reduced lamina, sparse hairiness and large cortical region.4) wasteland; **j** Hospital-greatly thicker leaves with reduced lamina, enlarge cortical parenchyma and vascular region, **k** Khanpur Nawan kot-thick leaf with narrow lamina, enlarge cortical region and vascular bundles area, **l** Model Town-thick leaf with narrow lamina and partially crushed cortical region, enlarged vascular region

**Fig. 5:**
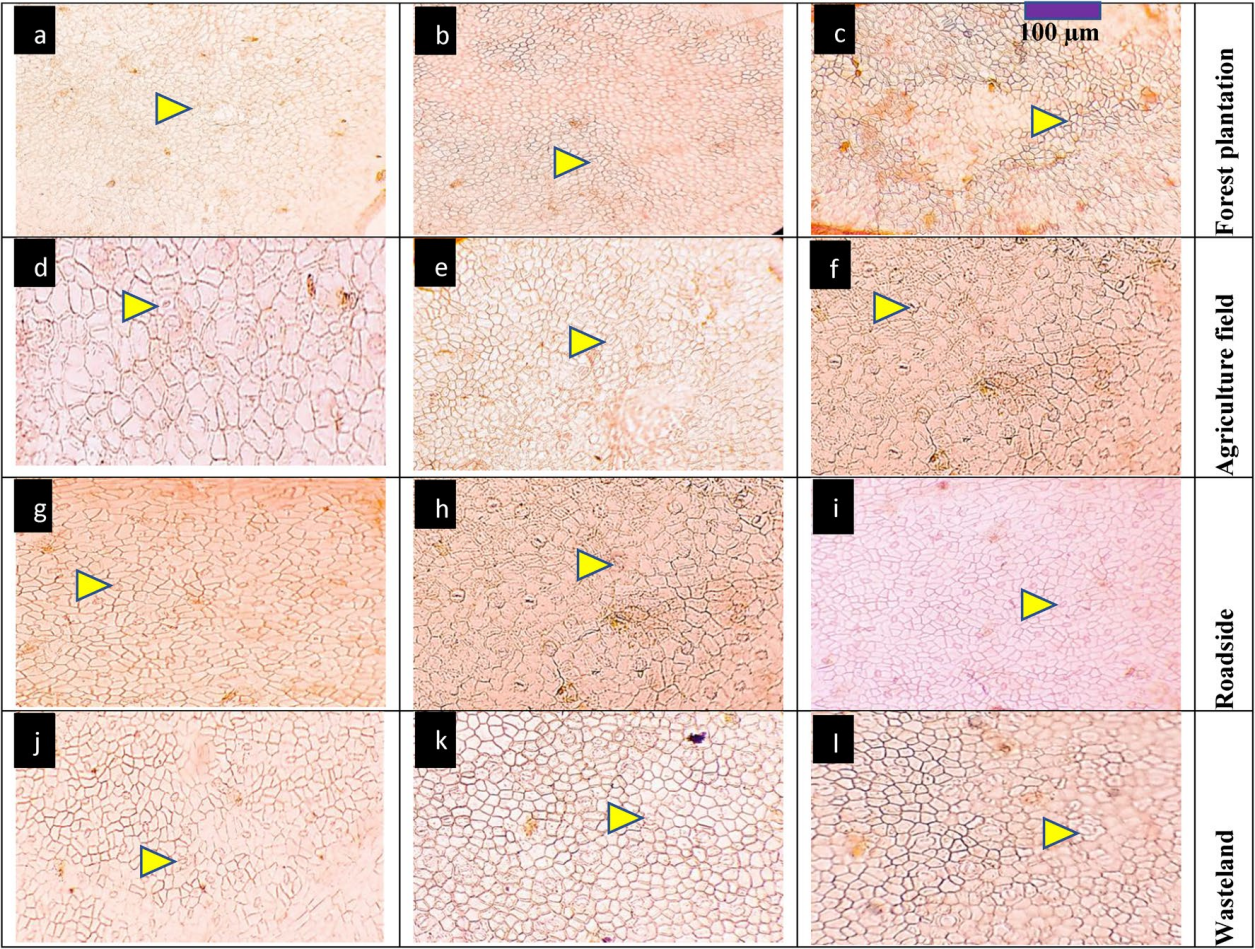
Epidermal transvers sections of *Conocarpus erectus* populations collected from the Punjab province (Mx = 40X; n = 6). Description: 1) forest plantation; **a** Chak 1- stomata small, narrower and deeply seated in epidermis, **b** Chak 4-small, narrower and sparsely arranged stomata, **c** Chak 96-stomata large and numerous with sparse alignment. 2) agriculture field; **d** Basti Sheikh-stomata few and small with sparse arrangement, **e** Chak 2P-stomata few and small with irregular alignment, **f** Dari Sanjhi-very large and numerous with irregular alignment. 3) roadsides; **g** Dera Bibi Sughra-very lare stomata with irregular arrangement, **h** Dhoop Sari-stomata very small and deeply seated in epidermis, **i** Flood colony-few and large stomata with irregular arrangement. 4)wasteland; **j** Hospital-stomata large and numerous with sparse alignment, **k** Khanpur Nawan kot-very few and small stomata with irregular arrangement, **l** Model Town-very enlarge stomata with regular alignment

In forest plantation habitats, such as C1, leaf anatomy showed thicker midribs and cortical layers (Table 4, Fig. 4). C1 had the maximum midrib thickness, along with the largest cortical cells, indicating robust leaf structure. However, populations like C9 displayed thinner epidermal layers and narrower metaxylem vessels, highlighting some variation within forest plantation habitats. Agriculture field populations such as BAS, C2, and DAS had moderate midrib thickness, with BAS showing the thinnest. C2 and BAS also had larger cortical cells and phloem areas. DAS possessed the largest vascular bundles and wider metaxylem vessels, indicating efficient vascular development. Stomatal density varied, with C1 from an agriculture field showing large stomata, whereas populations like DBS had smaller stomatal areas. Populations growing along roadsides, including FLC and DBS, showed moderate midrib and cortical thickness. DBS had smaller vascular bundles and narrower metaxylem vessels, reflecting lower water transport capacity compared to other habitats. Roadside populations also exhibited moderate stomatal numbers, with DBS showing the smallest stomatal area. Wasteland populations like MOT and KNK showed significant differences. MOT had the thickest lamina and moderate cortical thickness. KNK showed the smallest cortical cells and the thinnest metaxylem vessels, suggesting lower structural support. Stomatal numbers in wasteland populations were relatively high, though KNK displayed smaller stomata (Table 4, Fig. 5). The population HOP showed the thinnest lamina and narrow metaxylem vessels. These populations also exhibited reduced epidermal thickness and lower vascular bundle areas. HOP had one of the lowest stomatal densities, reflecting an adaptation to less favorable growing conditions.

## Multivariate analysis

### Principal component analysis (PCA)

PCA demonstrated a significant relationship between soil physicochemical characteristics and attributes of *C*. *erectus* populations.

**Fig. 6:**
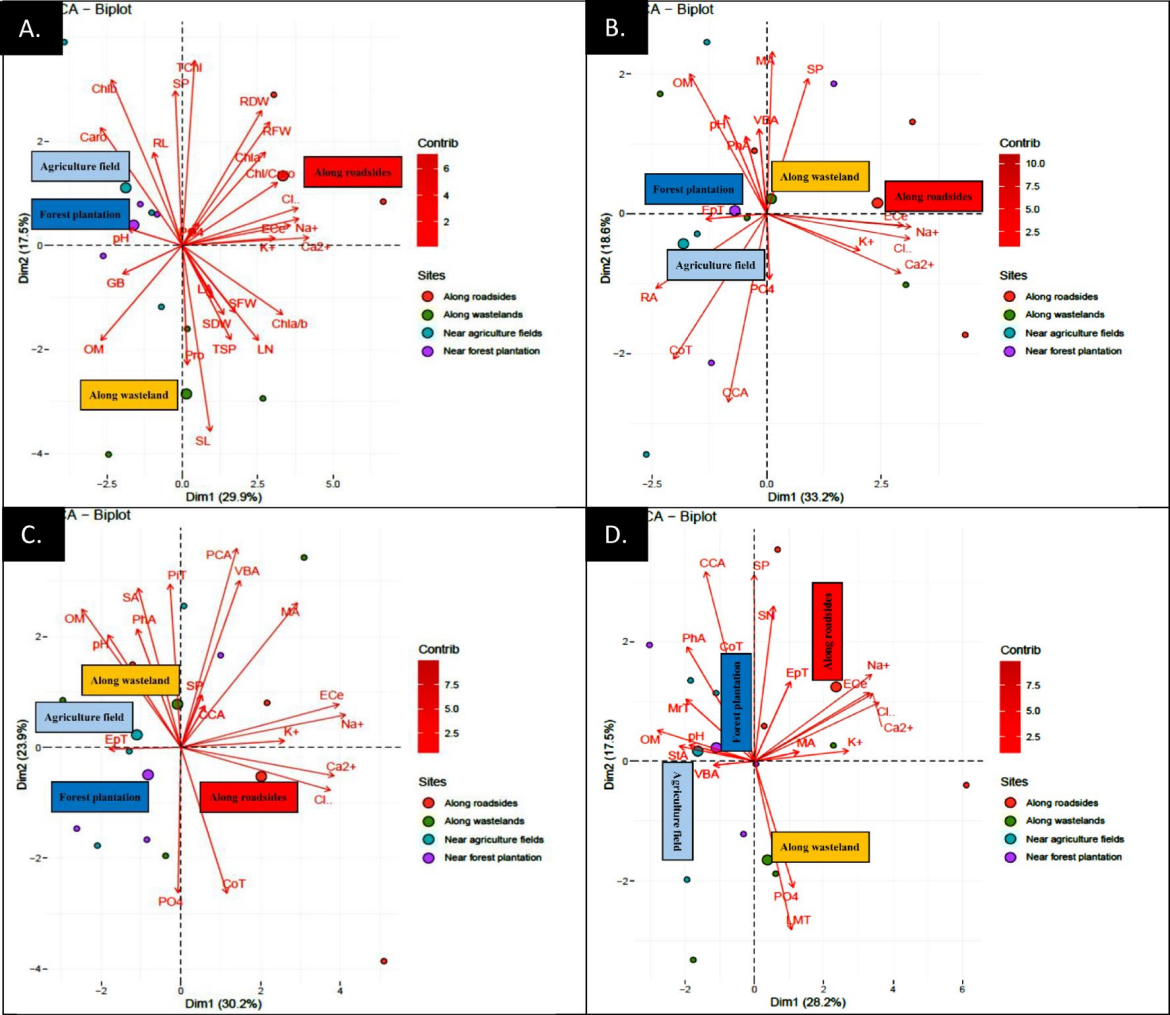
Principle component analysis (PCA) showing influence of soil physicochemical characteristics on **A** growth attributes and physiological attributes, **B** root anatomy, **C** stem anatomy and **D** leaf anatomical features of *Conocarpus erectus* from Punjab province

Along roadsides, traits such as RFW, RDW, SFW, Chl a, Chl b, TChl, pH, RL, and SDW exhibit strong associations with the plants in this habitat. In contrast, in wasteland habitats, traits like SL and Pro show weaker associations (Fig. 6A). VBA and MA exhibit associations with SP under wasteland conditions, while RA and CoT are associated with PO4 in agricultural field habitats (Fig. 6B). The wasteland region is associated with SA, PhA, PT, pH, and OM, while the roadside habitat is linked with CoT, Cl, and Ca (Fig. 6C). EpT and MA are closely associated with ECe and Na in roadside habitats, while CoT, PhA, SP, and CCA are key contributors in forest plantation habitats. Parameters such as MrT, StA, VBA, OM, and pH are associated with agricultural field habitats (Fig. 6D).

### Clustered heatmaps

**Fig. 7:**
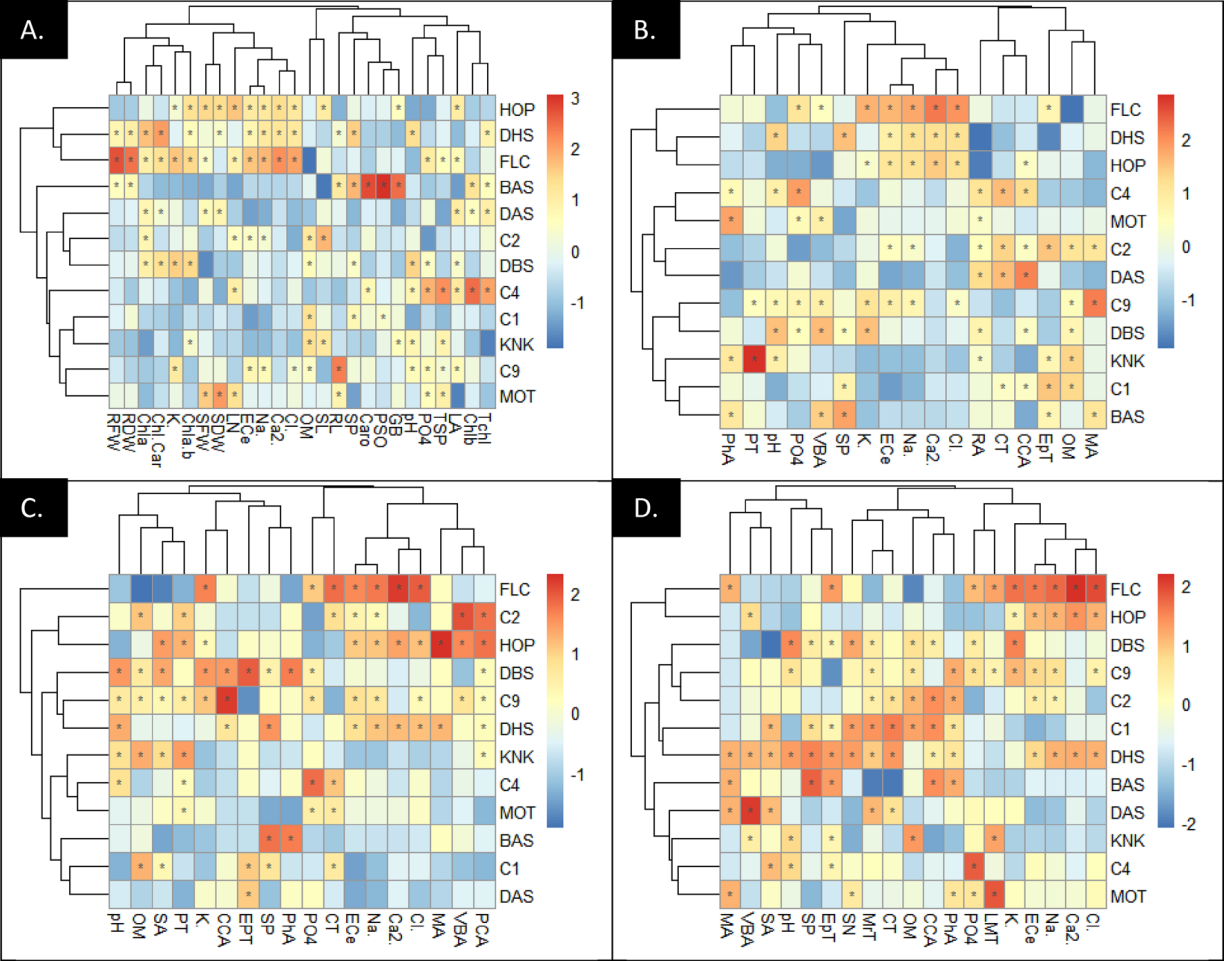
Clustered heatmaps showing association of soil physicochemical features with **A** morphology and physiology, **B** root anatomy, **C** stem anatomy and **D** leaf anatomical characteristics of *Conocarpus erectus* from Punjab province

Clustered heatmaps were constructed among soil physicochemical characteristics and species variables to assess their relationship under different habitats (Fig. 7A). The LN, SFW, SDW and Chlb showed strong association and clustering in FLC habitat, while the Tchl, Chlb, LA and TSP showed association under C4 habitat. The soil physiochemical features showed strong association and clustering with root anatomical features as given in Fig. 7B. A strong association was found among OM:EpT, SP:VBA and CCA:CT. a significant negative association was observed among pH:PT and MA:OM. In the case of soil physicochemical and stem anatomical heatmaps (Fig. 7C), the ECe:CT, CCA:K and SP:PhA showed very strong relation, whereas the MA:Cl, CT:PO_4_ and SA:OM showed negative correlation. In the relationship of soil attributes and leaf anatomical features (Fig. 7D), the CCA, OM, CT, MrT and SN showed strong association and clustering in C1 habitat, while the EpT, SP, pH, SA, VBA and MA showed very close association under DHS habitat.

## Discussion

**Fig. 8:**
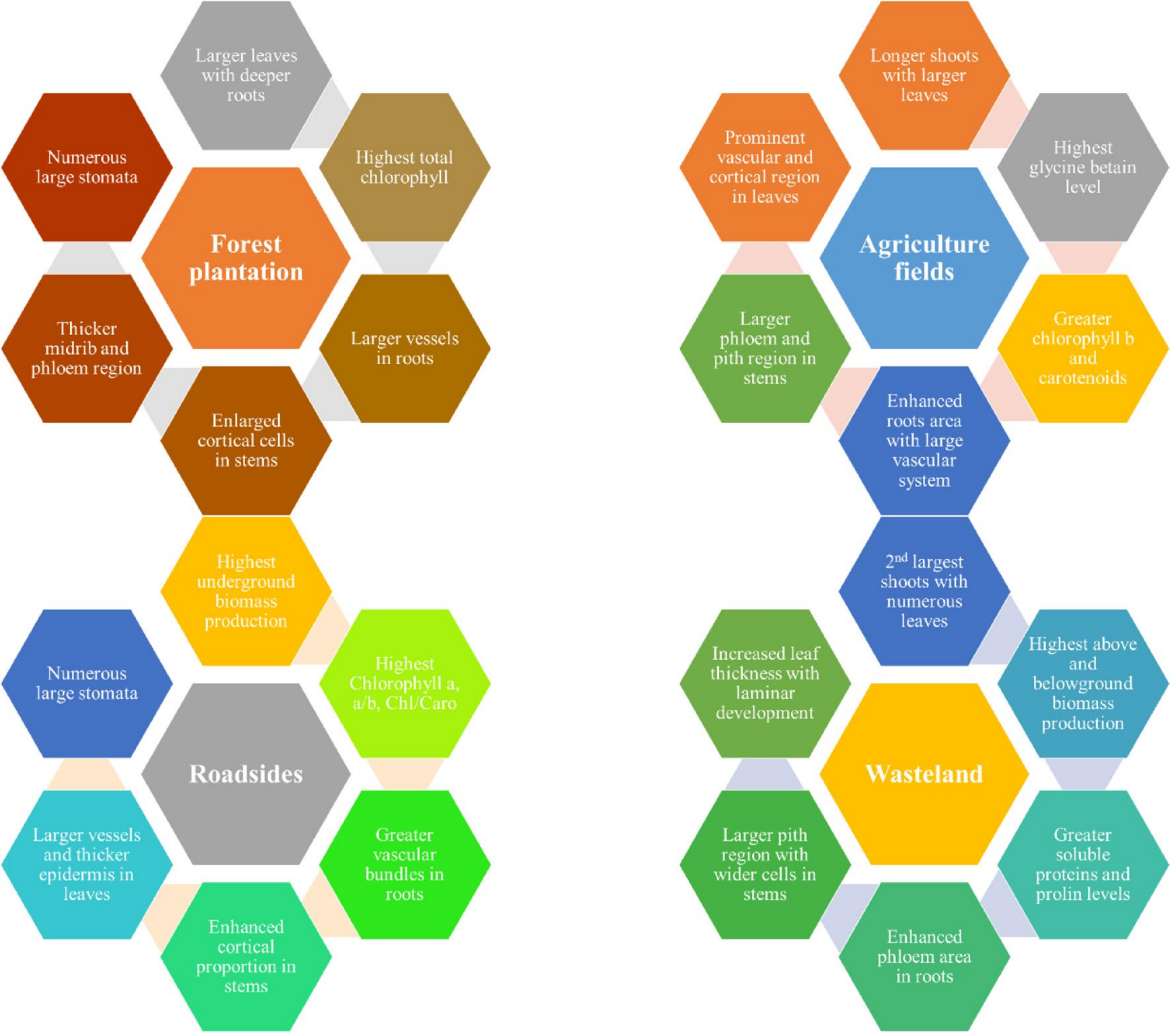
Adaptive component of *Conocarpus erectus* populations in response to diverse habitats of Punjab, Pakistan

Biotic and abiotic factors interact differently with dominant and non-dominant plants, as dominants often modify the environment for non-dominants. If dominant plants compete strongly, they deplete resources, restricting non-dominants to narrower niches. Alternatively, if dominants are limited by environmental constraints, they may reduce stressors, benefiting non-dominants. These dynamics can alter interactions among non-dominant species and may result in greater phylogenetic disparity between dominant and non-dominant species (Arnillas et al. 2021). Environmental heterogeneity is considered as major factor influencing species richness gradients. The expansion of available niche space, the provision of refuges, and opportunities for isolation and divergent adaptation are believed to promote species coexistence, persistence, and diversification (Stein and Kreft 2015). Naturally adapted populations or ecotypes usually showed various structural and functional alteration to climatic fluctuation for their growth regulation and survival under variable climatic conditions (Pandey et al. 2017; Khalid et al. 2020). They adopt specific anatomical adaptation to endure adverse environmental conditions such as stomata oriented in crept, thicker cuticle and epidermis, deep penetration of roots, and great proportion of storage tissues (Bibi et al. 2015). Populations of *C. erectus* were sampled from diverse habitats of Punjab province to investigate the alteration in growth performance, microstructural and functional attributes which makes this species more adoptive in heterogenic environments (Fig. 8).

### Structural and functional responses of plants

The populations near forest plantations exhibited distinct modifications in growth, physiological, and anatomical traits, particularly in response to their specific environments. For instance, the C9 population, located near a palm field, developed larger and more numerous roots, a significant ecological adaptation (Pessarakli 2015). In challenging climatic conditions, these longer roots allow plants to access water from deeper soil layers, aiding in drought tolerance (Abohassan et al. 2010; Gao et al. 2016). The C4 population, situated near a nursery, displayed large and narrow leaves, which likely enhance both photosynthetic efficiency and water-use efficiency by reducing transpiration (Medrano et al. 2015). Additionally, this population accumulated higher chlorophyll content, crucial for improving photosynthetic capacity under stress (Batool et al. 2013; Gu et al. 2017). Distinct anatomical adaptations were also observed among populations from artificial forests, closely related to their specific habitats and soil conditions. For example, the C1 population from an artificial forest developed thicker epidermis in the roots, a thicker midrib, enlarged cortical thickness, and more numerous stomata in the leaves. These adaptations are essential for preventing root damage due to soil compaction, improving water conservation, and enhancing water transport in hot and dry environments (Flowers & Colmer 2015; Kadam et al. 2015). The C9 population near the palm area also showed widened metaxylem vessels in the roots and expanded phloem areas in the leaves, both of which are critical for efficient water movement and the accumulation of photoassimilates throughout the plant (Nawaz et al. 2013). These modifications reflect the populations' ability to adapt to specific environmental stresses, enhancing their survival and performance in diverse habitats (Iqbal et al. 2023).

The populations from agricultural fields (BAS from cotton field, C2 from sugarcane field, DAS along rice field) exhibited distinct adaptations in their structural and functional features, which are closely linked to their specific habitats. A significant increase in growth parameters such as shoot length and leaf area was observed in these populations. This aligns with previous findings that populations from stressful environments tend to show stimulated growth when relocated to more favorable conditions, whereas populations from non-stressed habitats maintain normal growth (Abou-Leila et al. 2012; Hu et al. 2015). Furthermore, these populations demonstrated high levels of organic osmolyte accumulation, particularly glycine betaine, which plays a crucial role in osmoregulation and helps mitigate environmental stress (El-Mahrouk et al. 2010; Anjum et al. 2017). In particular, the BAS population from the cotton field showed an increase in photosynthetic pigments such as chlorophyll b and total chlorophyll. This suggests that the population thrives in a relatively favorable environment, with increased pigment levels supporting higher photosynthetic efficiency (Redha et al. 2012). Additionally, BAS exhibited larger vascular bundles in the root, greater epidermal thickness, and increased phloem area in the stem, all of which are critical for efficient water transport, surface protection, and enhanced photoassimilate translocation (Hegazy et al. 2008; Corrêa et al. 2016). The C2 population from the sugarcane field presented increased cortical thickness and cellular area in the root, alongside expanded vascular bundle and metaxylem areas in the leaf. These modifications are indicative of improved water and nutrient transport, supporting enhanced survival in moisture-sufficient environments (Smith et al. 2013). Another key adaptation in the C2 population was the development of larger stomata, which are crucial for optimizing photosynthetic efficiency while regulating water loss through transpiration (Akram et al. 2013). These findings underscore the importance of habitat-specific adaptations in agricultural field populations, where structural changes such as increased vascular bundle size, cortical thickness, and epidermal modifications contribute to the plants' ability to thrive under variable environmental conditions.

The roadside populations exhibited notable adaptations in root biomass and chlorophyll content, surpassing other populations. Biomass production is a crucial indicator of a population's survival and tolerance to varying environmental conditions (Shirazi et al. 2006; Zokaee-Khosroshahi et al. 2014). For instance, the FLC population (along the roadside) showed substantial growth potential, with an increase in root biomass through longer and more numerous roots. This is consistent with previous studies, which have linked enhanced root biomass to drought tolerance, as seen in lentils (Talukdar 2013) and *C*. *dactylon* (Ye et al. 2015). The DHS population displayed adaptations such as enhanced mesophyll tissues and increased stomatal size and number. These modifications are key to improving gas exchange and photosynthetic efficiency in dry environments (Melotto et al. 2017). Such structural changes allow the species to cope with water stress by maximizing carbon fixation while limiting water loss. Similarly, the DBS population demonstrated notable modifications, including a multilayered epidermis and a thickened phloem region in the stem. The epidermis plays a critical protective role, shielding internal tissues from environmental stressors, such as desiccation, particularly in arid conditions (Iqbal et al. 2022a). A thicker epidermis acts as a barrier against water loss and protects against tissue dehydration. These findings demonstrate the diverse range of structural and physiological adaptations employed by different populations to survive in both dry and saline environments. Root biomass, stomatal adjustments, and vascular modifications all contribute to enhancing the resilience and survival potential of these species under varying environmental stresses.

The wasteland populations exhibited decisive morpho-anatomical and physiological adaptations to cope with harsh environmental conditions. The population growing along the wasteland (MOT) showed significant increases in shoot biomass, total soluble protein content, and the development of succulent leaves with thicker midribs and laminae. The enlargement of metaxylem vessels in both the roots and leaves also played a key role in improving water and nutrient translocation, enhancing overall plant growth and resilience (Zhao et al. 2020; Tufail et al. 2020). Biomass production, along with elevated levels of soluble proteins, suggests the population’s capacity to adapt to environmental stress by bolstering growth and solute transport. Populations thriving in degraded lands, such as HOP, faced a dual challenge: ionic toxicity and physiological drought. These populations developed key structural features, including thicker stem areas, enlarged metaxylem vessels, and increased pith thickness and cell area. These modifications are crucial for improving water storage and transport, which enhances the plants' ecological fitness in saline habitats. Stem cross-sectional area expansion is particularly advantageous in resisting abiotic stress, as it increases the water storage capacity of the cortex and pith, ensuring sustained growth under challenging conditions (Naz et al. 2010; Corrêa et al. 2016). Additionally, the reduction in epidermal cell area along salinity gradients, as observed in several populations, likely reflects a strategic adaptation to reduce water loss in arid environments (Naz et al. 2016; Kaleem et al. 2024). Increasing hypodermal cell area and thickness is another crucial adaptation in desert plants, enabling them to conserve maximum water when subjected to prolonged drought or physiological drought conditions (Parida et al. 2016). Conversely, a decrease in stem cortical thickness was observed under higher salinity levels, which may represent tissue deterioration triggered by salinity stress (Eisa 2017). This reduction can be beneficial by curtailing unnecessary growth and reducing energy expenditure. Furthermore, a reduction in metaxylem and phloem area under high salinity conditions leads to decreased hydraulic activity, safeguarding the plants' conducting vessels (Junghans et al. 2006). However, this also limits the translocation of nutrients to aerial parts or back to underground organs, which can restrict growth in high-salinity environments. Sclerenchyma bundles and cell areas increased with salinity in all populations, as the development of sclerenchyma tissue provides essential mechanical strength during salt and osmotic stress (Alvarez et al., 2008; Iqbal et al. 2021). Furthermore, increases in pith area and thickness were particularly beneficial for plants in saline environments, enhancing their succulence, a critical trait for desert-dwelling species (Parida et al. 2016).

### Adaptive mechanisms driving ecological success of *C. erectus*

The ecological success of *C. erectus* is driven by several key modifications that enhance its adaptability to challenging environments. Populations of *C. erectus* in wasteland habitats exhibit significant increases in shoot biomass production, reflected in both fresh and dry weight, which indicates improved growth potential under stress. This is accompanied by an accumulation of total soluble proteins, crucial for osmoregulation in saline conditions, allowing the plant to mitigate environmental stresses (Iqbal et al. 2024). The development of succulent leaves, characterized by increased midrib and lamina thickness, further enhances water storage capacity, enabling the plant to withstand drought conditions (Ameer et al. 2023). Additionally, the enlargement of metaxylem vessels in both roots and leaves facilitates efficient translocation of water and nutrients, promoting overall growth. Structural modifications, such as increased stem cross-sectional area and enhanced storage parenchyma (cortex and pith), contribute to the water storage capacity essential for coping with drought and salinity (Irshad et al. 2024).

Moreover, the thickening of epidermal and hypodermal tissues acts as a protective barrier, conserving moisture and reducing water loss during stressful periods (Asghar et al. 2023). Adaptive changes in mesophyll structure, particularly the proportion of palisade and spongy mesophyll cells, ensure that *C. erectus* maintains photosynthetic efficiency and effectively manages ionic toxicity from accumulated salts (Basharat et al. 2024a, b). The development of sclerenchyma tissue also provides mechanical strength during salt and osmotic stress, contributing to the plant's structural integrity (Iqbal et al. 2022b). Collectively, these modifications enhance the ecological fitness of *C. erectus*, allowing it to thrive in environments characterized by salinity, drought, and other abiotic challenges.

## Conclusion

The populations of *C. erectus* exhibited remarkable modifications related to growth, structural and functional attributes, enabling this species to thrive in a wide range of habitats. Structurally, it exhibits wider xylem vessels in roots, enlarged vascular bundles in stems and leaves, well-developed storage parenchyma (cortex and pith), sparse surface hairiness, small and numerous stomata, and thicker leaves. Functionally, it possesses higher contents of chlorophyll and organic osmolytes (TSP, Pro and GB). These modifications at the structural and functional levels significantly contribute to the ecological success of this species in agricultural lands, forest plantations, roadsides, and wastelands.

## Supplementary Information

**Additional file 1.**

## Data Availability

All relevant data are within the paper.
