# Supplementary figures and images for: Unveiling the ecological dominance of button mangrove (Conocarpus erectus L.) through microstructural and functional traits modifications across heterogenic environmental conditions
Source: Bot Stud. 2024 Nov 29;65:36. doi: 10.1186/s40529-024-00440-0 (PMC11604896; doi:10.1186/s40529-024-00440-0)

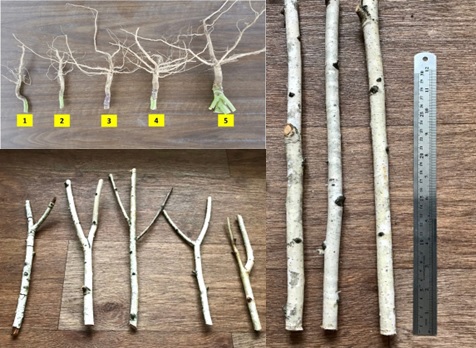

Supplement: Supplementary file 1 — Additional file 1. [file 40529_2024_440_MOESM1_ESM.jpg]
